# Postoperative Infection Following Hip Arthroscopy in Patients Receiving Preoperative Intra-articular Injections: A Systematic Review and Meta-Analysis

**DOI:** 10.7759/cureus.61649

**Published:** 2024-06-04

**Authors:** Sonia Aamer, Ilham Tokhi, Maaz Asim, Muzammil Akhtar, Daniel I Razick, Jimmy Wen, Trevor J Shelton

**Affiliations:** 1 Orthopedic Surgery, California Northstate University College of Medicine, Elk Grove, USA; 2 Surgery, California Northstate University College of Medicine, Elk Grove, USA; 3 Physical Medicine and Rehabilitation, California Northstate University College of Medicine, Elk Grove, USA; 4 Orthopedic Surgery, Utah Valley Orthopedics and Sports Medicine, Provo, USA

**Keywords:** hip surgery, outcomes, infection, intra-articular injection, hip arthroscopy

## Abstract

Intra-articular injections prior to hip arthroscopy are often used to diagnose and conservatively manage hip pathologies, such as femoroacetabular impingement, labral tears, and chondral lesions. As a diagnostic tool, the relief of hip pain following an intra-articular injection helps pinpoint the primary source of pain and assists surgeons in recommending arthroscopic intervention for underlying intra-articular pathologies. However, when injections are not sufficiently spaced apart in time prior to hip arthroscopy, there is an elevated risk of postoperative infection. This systematic review aims to assess whether preoperative intra-articular injections prior to hip arthroscopy are associated with an increased risk of postoperative infection and to determine the safety timeframe for administering such injections prior to the procedure. A comprehensive search was conducted in the PubMed, Embase, and Cochrane Library databases to identify studies examining the relationship between preoperative intra-articular injections and postoperative infection following hip arthroscopy. A meta-analysis was conducted to compare the risk of infection between patients who received injections prior to hip arthroscopy at varying intervals and those who did not receive any preoperative injections. Five studies were included (four level III and one level IV), which consisted of 58,576 patients (58.4% female). Injections administered anytime prior to hip arthroscopy posed a significantly higher risk of infection compared to no history of prior injections (risk ratio: 1.45, 95% confidence interval: 1.14-1.85, P = 0.003). However, upon subanalysis, the risk of infection was significantly higher among patients who received injections within three months prior to hip arthroscopy compared to those who did not receive injections (risk ratio: 1.55, 95% confidence interval: 1.19-2.01, P = 0.001). Additionally, no significant difference in infection risk was observed when injections were administered more than three months before hip arthroscopy compared to no injections (risk ratio: 1.05, 95% confidence interval: 0.56-1.99, P = 0.87). The findings suggest that patients undergoing hip arthroscopy who have previously received intra-articular injections may face a statistically higher risk of postoperative infection, particularly when the injection is administered within three months prior to hip arthroscopy. Consequently, surgeons should exercise caution and avoid administering intra-articular injections to patients scheduled for hip arthroscopy within the subsequent three months to mitigate the increased risk of infection.

## Introduction and background

Hip arthroscopy has increasingly been utilized over the past few decades for addressing intra-articular hip pathologies, such as femoroacetabular impingement, labral tears, and chondral lesions, yielding favorable outcomes. Prior to hip arthroscopy, intra-articular injections are often used by orthopedic surgeons as both diagnostic tools and therapeutic nonoperative treatments for hip pain unresponsive to other conservative management methods. As a diagnostic tool, relief of hip pain following an intra-articular injection helps isolate the primary pain source and guides surgeons in recommending arthroscopic intervention for underlying intra-articular pathology. Conversely, a lack of pain relief following an intra-articular injection may indicate extra-articular sources of hip pain [1-3]. An increasing number of surgeons and institutions now incorporate intra-articular injections to guide clinical decision-making in managing hip pain.

Despite their widespread use for diagnostic and therapeutic purposes, recent studies have reported an increased risk of infection when intra-articular injections are administered too close to elective surgeries, such as hip arthroscopy, generally within three months prior [4-8]. For example, a recent meta-analysis involving over 300,000 patients found that administering preoperative intra-articular steroid injections within three months of total knee arthroplasty (TKA) significantly increased the odds of periprosthetic joint infection (PJI) (P < 0.01) [9]. Similarly, another study indicated a significantly higher risk of PJI when intra-articular injections were given within three months prior to total shoulder arthroplasty (TSA), with elevated odds of infection at both three (P = 0.007) and six months (P = 0.001) postoperatively. However, when injections were administered three to 12 months before TSA, the odds of PJI were comparable to those without preoperative injections [10]. A systematic review of eight studies in 2020 evaluating the risk of infection in patients undergoing rotator cuff repair who were previously administered corticosteroid injections reported significantly higher odds of infection when injections were given within six months of the surgery or if ≥ 2 injections were administered within a year [11].

With the increasing use of intra-articular hip injections to guide clinical decision-making and determine candidacy for hip arthroscopy, it is crucial to assess the associated risks. Therefore, the primary objective of this systematic review is to evaluate whether preoperative intra-articular injections increase the risk of postoperative infection following hip arthroscopy. The secondary objective is to determine the safe interval for administering these injections before hip arthroscopy. We hypothesize that administering intra-articular injections closer to the time of hip arthroscopy increases the risk of postoperative infection.

## Review

Methods

A search following guidelines established by the Preferred Reporting Items for Systematic Reviews and Meta-Analyses (PRISMA) was performed in three databases in April 2024: PubMed, Embase, and Scopus. Two authors identified all articles included in the study. The query was performed utilizing the Boolean search phrase “(hip AND arthroscop* AND injection).” There were no restrictions set to the search. Studies were included if they reported on postoperative infection in patients who received intra-articular injections prior to hip arthroscopy. Exclusion criteria included case reports, review articles, conference abstracts, studies performed in animals, articles not in English, expert opinions, letters to editors, and studies in which outcomes pertaining to preoperative intra-articular injections prior to hip arthroscopy were not specified.

The titles and abstracts of all studies were independently reviewed by two reviewers using the predetermined eligibility criteria. If they were not unanimous in their decision to include or exclude a study, a third reviewer was consulted. Next, the full text of select articles was independently reviewed by two reviewers, and again, if the reviewers were not unanimous in their decision, a third reviewer was consulted. All included articles underwent rigorous reference search to determine whether additional studies could be added to the systematic review. Study variables extracted from each article included study characteristics, patient demographic information, time between preoperative injection and subsequent hip arthroscopy, number of injections, and incidence of postoperative infection and revision.

The methodological quality of studies was assessed using the methodological index for non-randomized studies (MINORS) checklist. The MINORS items are scored 0 (not reported), 1 (reported but inadequate), or 2 (reported and adequate), with a maximum possible score of 16 for non-comparative studies (from eight categories) and 24 for comparative studies (from 12 categories). Two authors scored each article in the systematic review. Each author scored the article individually before reviewing their scores, and any discrepancies were resolved by re-reviewing the articles until a unanimous consensus was met.

Descriptive statistics (mean, percentage, standard deviation, range, median) are reported in this study when applicable and when available. A meta-analysis was performed to compare the risk of infection between patients who received injections prior to hip arthroscopy at various time points with those who received no prior injections. Forest plots were generated to depict overall significance. A P-value of less than 0.05 was considered statistically significant.

Results

**Figure 1:**
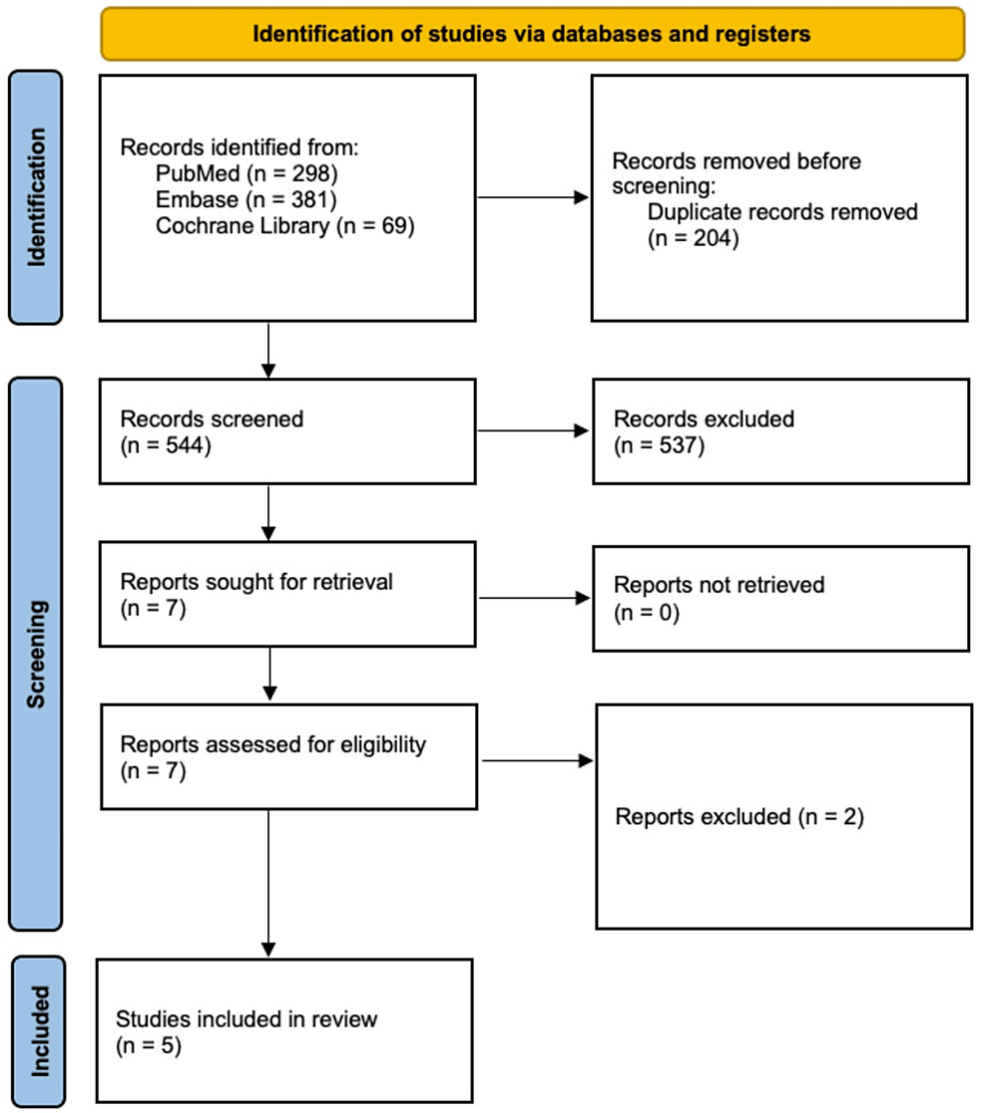
PRISMA flow chart depicting the article selection process PRISMA, Preferred Reporting Items for Systematic Reviews and Meta-Analyses

Upon the initial search of the PubMed, Embase, and Cochrane Library databases, 748 studies were identified, with 204 duplicates removed. The remaining 544 studies underwent full title and abstract review, resulting in the removal of 537 studies based on our predetermined exclusion criteria. Seven studies underwent full-text review to determine eligibility. Two studies were excluded for not reporting on infection following hip arthroscopy in patients who received preoperative intra-articular injections or due to overlapping study periods with multiple studies from the same senior author and institution. Consequently, five studies were included in this systematic review. The PRISMA flow diagram illustrating our search strategy and article selection process is depicted in Figure 1.

All five studies had a retrospective design, with four of the five studies utilizing national administrative databases. Four studies had a level of evidence of III, while one study had a level of evidence of IV. In total, there were 58,576 patients (58.4% female) across the five studies. Table 1 provides a summary of the study design, MINORS score, number of patients, and main findings from each study.

**Table 1:** Study characteristics and main findings HA, hip arthroscopy; CSI, corticosteroid; MINORS, methodological index for non-randomized studies; OA, osteoarthritis; THA, total hip arthroplasty; FL, fluoroscopic; US, ultrasound

Author	Study Design	MINORS Score	Patients (Female/Male)	Main Findings
Varady et al. [4]	Patients were grouped into a control group which received no injections, and two groups which received an injection within 0-3 and 3-12 months of HA. Patients who received injections under FL vs. US guidance were evaluated separately. Postoperative infection was evaluated within 6 months of the HA procedure.	19/24	0-3 months: 1262 (368/894)	For FL-guided injection, there was no significant difference in infection rate between the 0-3 month vs. Control group (P > 0.999) and the 3-12 month vs. Control group (P = 0.74).
					3-12 months: 1014 (340/674)
			For US-guided injection, there was no significant difference in infection rate between the 0-3 month vs. Control group (P = 0.76) and the 3-12 month vs. Control group (P > 0.999).		
				Control: 15,711 (5814/9897)
					Wang et al. [5]	Patients were grouped into a control group which received no injections, and three groups which received an injection within 0-3, 3-6, and 6-12 months of HA. Patients who were privately insured and those who were covered by Medicare were evaluated separately. Postoperative infection was evaluated within 6 months of the HA procedure.	18/24	0-3 months: 339 (230/109)	For privately insured patients, the 0-3 month vs. control group had a significantly higher infection rate (P < 0.001), however, both the 3-6 month and 6-12 month groups compared to the control group did not have significant differences in infection rate (P = 0.285 and 0.396, respectively).
			3-6 months: 249 (168/81)						
6-12 months: 186 (131/55)	For Medicare patients, the 0-3 month vs. control group had a significantly higher infection rate (P < 0.037); however, both the 3-6 month and 6-12 month groups compared to the control group did not have significant differences in infection rate (P = 0.172 and 0.195, respectively).						
Control: 6846 (4295/2551)							
Surucu et al. [8]	Patients were grouped in a control group which received no injections, and three groups which received an injection within 0-4, 4-8, and 8-12 weeks of HA.	18/24	0-4 weeks: 3579 (2611/968)	Patients in the 0-4 week group had significantly greater odds of infection compared to the control group (P = 0.0005). Patients in both the 4-8 and 8-12 week groups had similar odds of infection compared to the control group (P = 0.1543 and 0.1352, respectively). Additionally, patients who received an injection vs. those who did not had a significantly higher rate of infection (P < 0.0001).
			4-8 weeks: 4759 (3426/1333)	
			8-12 weeks: 4052 (2383/1669)	
			Control: 12,390 (9012/3387)	
Johnson et al. [7]	Patients were grouped into a control group which received no injections and an injection group which received an injection within a year of HA. Injections were administered under US or FL guidance. The injection group was subdivided into those who received CSI or local anesthetic injections. Patients were evaluated at 1 and 5 years after HA.	20/24	Injection: 6511 (4488/2023); (CSI (3739) and Local Anesthetic (2749))	Any injection vs. control group had significantly higher odds of repeat HA at 1 and 5 years (P < 0.001 for both), but not THA, infection, or new onset OA.
				CSI vs. control group had significantly higher odds of repeat HA at 1 and 5 years (P < 0.001 for both), but not THA, infection, or new onset OA.
			Control: 1178 (563/615)	Local anesthetic vs. control group had significantly lower odds of repeat HA at 1 year (P < 0.001) but not at 5 years (P = 0.361). Local anesthetic vs. control group did not have significantly higher odds of THA, infection, or new onset OA at 1 or 5 years.
				CSI vs. local anesthetic had significantly greater odds of repeat HA at 1 and 5 years (P < 0.001 for both), but not THA, infection, or new onset OA.
Byrd et al. [6]	Only patients who received injections within three months of HA were included. Injections were administered under US guidance and consisted of 1 mL (40 mg) methylprednisolone, 4 mL 1% lidocaine, and 4 mL 0.25% bupivacaine.	7/16	500 (388/112)	There were zero postoperative cases of infection.

Three studies compared the rates or odds of postoperative infection in hip arthroscopy patients who received intra-articular injections at various time points prior to hip arthroscopy with those who received no prior injections. Varady et al. [4] found that, when compared to the control group, there was no significant difference in the rate of infection when either fluoroscopic or ultrasound-guided injections were administered within zero to three or three to 12 months of hip arthroscopy. Conversely, Wang et al. [5] reported a significantly higher infection rate in patients receiving injections within zero to three months prior to hip arthroscopy, while those receiving injections three to six or six to 12 months before surgery had similar infection rates to the control group. Surucu et al. [8] assessed infection odds at smaller intervals and found significantly greater odds of infection in patients receiving injections zero to four weeks before surgery, but similar odds for those receiving injections four to eight or eight to 12 weeks before surgery compared to the control group.

**Figure 2:**
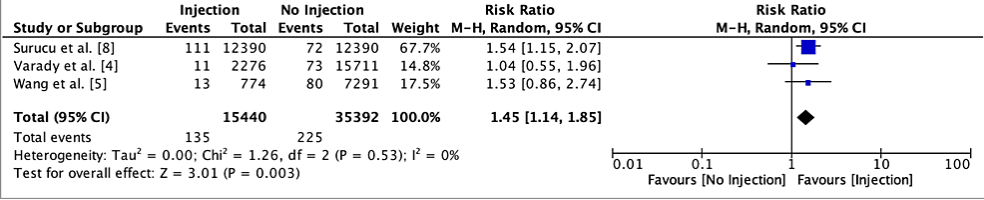
Forest plot depicting the risk of infection in patients receiving an injection any time prior to hip arthroscopy versus those receiving no injection Refs. [4,5,8]

**Figure 3:**
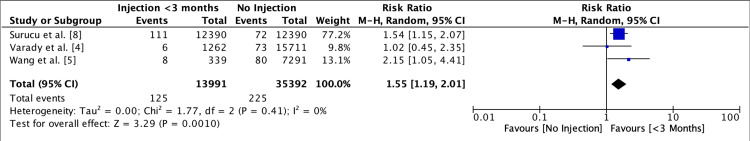
Forest plot depicting the risk of infection in patients receiving no prior injection versus those receiving an injection within three months of hip arthroscopy

**Figure 4:**
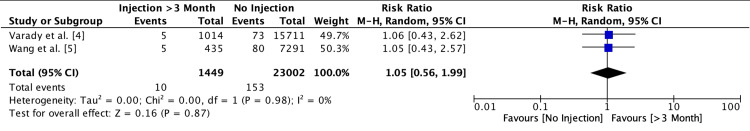
Forest plot depicting the risk of infection in patients receiving no prior injection versus those receiving an injection more than three months prior to hip arthroscopy Refs. [4,5,8]

**Figure 5:**
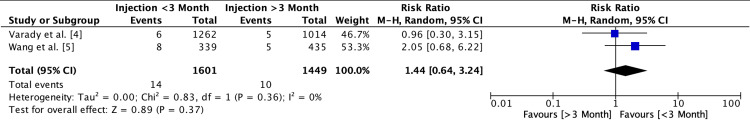
Forest plot depicting the risk of infection in patients receiving an injection within three months versus greater than three months prior to hip arthroscopy Refs. [4,5]

The risk of infection was significantly higher in patients receiving any injection prior to hip arthroscopy compared to those with no injection (risk ratio = 1.45; 95% confidence interval = 1.14-1.85; P = 0.003; Figure 2). Specifically, the risk was higher in the group receiving injections within three months prior to surgery (risk ratio = 1.55; 95% confidence interval = 1.19-2.01; P = 0.001; Figure 3). There was no significant difference in infection risk for patients receiving injections more than three months prior to surgery compared to those with no injection (risk ratio = 1.05; 95% confidence interval = 0.56-1.99; P = 0.87; Figure 4). Similarly, the infection risk was not significantly different between patients receiving injections within versus greater than three months before surgery (risk ratio = 1.44; 95% confidence interval = 0.64-3.24; P = 0.37; Figure 5).

Johnson et al. [7] compared the odds of repeat hip arthroscopy, total hip arthroplasty (THA), infection, and new-onset osteoarthritis (OA) at one and five years following hip arthroscopy among patients receiving a prior corticosteroid injection, local anesthetic injection, or no injection. They found no significantly greater odds of infection, THA, or new-onset OA between any injection versus no injection, corticosteroid injection versus no injection, local anesthetic injection versus no injection, and corticosteroid injection versus local anesthetic injection at both one and five years post surgery. However, corticosteroid injections were associated with significantly greater odds of repeat hip arthroscopy compared to local anesthetic injections at both one and five years.

Byrd et al. [6] conducted a retrospective review of 500 consecutive hip arthroscopy patients who had received an intra-articular injection within three months prior to surgery. None of these patients developed postoperative surgical site infections.

Discussion

The primary findings of our study demonstrated that patients receiving an intra-articular injection within three months prior to hip arthroscopy had a significantly greater risk of postoperative infection, whereas those receiving an injection more than three months prior did not have a higher risk of infection. However, when comparing patients who received an injection within three months to those who received one more than three months prior, the risk of postoperative infection was similar. Additionally, patients receiving an injection at any time point prior to hip arthroscopy had a significantly higher risk of infection compared to those who received no prior injections.

The results of our meta-analysis align with findings from similar studies on other orthopedic procedures, such as joint arthroplasty and rotator cuff repair. Albanese et al. in their meta-analysis on the risk of PJI following total joint arthroplasty in patients who received preoperative corticosteroid injections found no association between PJI and TKA. However, they observed a statistically higher risk of PJI (odds ratio: 1.2, 95% confidence interval: 1.058-1.347, P = 0.045) if injections were administered within three months prior to THA [12]. Similarly, Lucenti et al., in their systematic review on infection risk in patients who received corticosteroid injections before TSA or shoulder arthroscopy, determined that the risk of postoperative infection was greater when injections were given within three months of surgery [13].

It is important to emphasize that the timing of the injection is not the sole risk factor for postoperative infection. Other factors, including obesity, sex, smoking, and alcohol use, also impact outcomes. Our systematic review included three studies for meta-analysis, which had variations in baseline patient demographics, comorbidities, and additional risk factors. These differences, along with the timing of the injection, may have contributed to postoperative infection rates.

This study should be considered in the context of its limitations. First, based on our strict inclusion and exclusion criteria, we were limited to only five included studies in the systematic review, of which only three were able to be included in the meta-analysis. Though the meta-analysis encompassed a large number of patients, since they were derived from only three studies, the generalizability of the results may be limited. Second, data on factors such as injection technique, concentration, volume, and contents were either incompletely presented or not provided by the included studies, introducing potential confounding bias. Finally, all included studies had a retrospective design. Although it would be pragmatically challenging to address the research question through studies with stronger designs, such as randomized controlled trials, the use of large administrative databases is warranted to provide more robust evidence.

## Conclusions

Patients undergoing hip arthroscopy who previously received intra-articular injections may face a statistically higher risk of developing postoperative infection, particularly when the injection is administered within three months prior to the procedure. Therefore, to mitigate the increased risk of infection, surgeons should avoid administering intra-articular injections to patients scheduled for hip arthroscopy within the subsequent three months.
